# Metal‐Dependent Effects and Crowding Robustness of *Pseudomonas fluorescens* Esterase I

**DOI:** 10.1002/cbic.70424

**Published:** 2026-06-07

**Authors:** Emmanouil Ntermanakis, Nikoleta Syngelaki, Alexandros Lyratzakis, Renia Fotiadou, Spyridoula Charova, Ioannis V. Pavlidis, Angeliki Giannouli

**Affiliations:** ^1^ Department of Chemistry University of Crete, Voutes Campus Heraklion Greece

**Keywords:** biocatalysis, catalytic site, copper, molecular crowders, zinc

## Abstract

Industrial biocatalysts are strongly influenced by their physicochemical environment, yet systematic studies on how metals and macromolecular crowding influence metal‐independent esterases remain scarce. Here, *Pseudomonas fluorescens* esterase I (PFE), an α/β‐hydrolase, is employed to evaluate how divalent cations and molecular crowding modulate catalysis and structural integrity. PFE retained >85% activity for most metals at 2 mM, demonstrating notable robustness. By contrast, Cu^2+^ and Zn^2+^ induced pronounced inhibition, with loss of activity at higher metal concentrations. Combined activity, EDTA recovery, dynamic light scattering, and native–PAGE experiments reveal Zn^2+^ and Cu^2+^ perturb PFE's structure, promoting structural heterogeneity and the formation of aggregates, whereas for Cu^2+^ computational predictions further support interactions with active site residues. Mg^2+^, Ca^2+^, Ba^2+^, Mn^2+^, Co^2+^, and Ni^2+^ exert weaker and variable effects. Unlike chemical perturbation, macro‐ and micromolecular crowding was idle to PFE's activity, despite increases in viscocity, underscoring resilience to physical crowding. Overall, PFE responses to metals are governed by metal‐specific coordination chemistry and distinct deactivation mechanisms, providing a framework for understanding esterase performance in metal‐rich and crowded environments and highlighting physicochemical tuning as a complementary strategy to protein engineering in biocatalysis.

## Graphical abstract

*Pseudomonas fluorescens* esterase I retains catalytic activity in metal‐rich and crowded environments, despite metal‐specific inhibitory effects. These findings highlight its robustness for biocatalytic applications in complex environments.

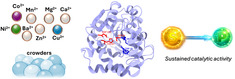

## 1 Introduction

Enzymes are quietly becoming the workhorses of modern chemistry, catalyzing reactions that once required harsh conditions and precious metals as catalysts [1]. Hydrolases, in particular, are valuable in industrial applications due to their high chemo‐ and enantio‐selectivity that enables greener routes to active pharmaceuticals, flavors, fragrances, and advanced materials [2]. There is growing recognition that the physicochemical environment of hydrolases, including metal ions and crowders, may critically modulate their structure and function in ways that matter to industrial biocatalysis. However, even for well‐established enzymes, two questions remain central for biocatalysis: (i) *How will the biocatalyst behave in non‐native conditions*, *e.g. in m*etal–*rich broth of a process stream or in media that mimic the crowded intracellular environment?* and (ii) *Can performance be tuned without another round of cloning evolution?*

Divalent metal cations such as Mg^2+^, Ca^2+^, Ba^2+^, Mn^2+^, Co^2+^, Ni^2+^, Cu^2+^, and Zn^2+^ may play a dual role. On one hand, they are ubiquitous in industrial feeds, as well as in ecological niches, where metal homeostasis shapes enzyme evolution and stress responses [3] and at the same time, they can stabilize a reactive conformation or drive protein aggregation depending on the ionic potential and coordination geometry [4, 5]. Spectroscopically derived kinetics of hydrolases and arylesterases have documented both metal‐dependent inhibition and activation [6, 7, 8]. However, a coherent picture of how a defined metal series affects a single well‐characterized esterase under bio‐catalytically relevant conditions is still missing. Particularly, it is still unclear whether metals act primarily through active‐site binding, global unfolding, or promote enzyme aggregation [1, 2, 9, 10].

Another important but often overlooked feature of biological environments is macromolecular crowding. Inside cells, enzymes function in highly crowded media, which can stabilize native conformations, alter dynamics and modulate diffusion of substrates and products [11, 12]. Macromolecular crowders are frequently invoked as “cell‐mimetic” handles to tune enzyme behavior, but their kinetic impact varies depending on specific interactions with the enzyme of interest and the solvent [13]. Typically, the activity and stability of hydrolases and related esterases are evaluated in dilute buffers, thus it remains unclear to what extent the enzyme is sensitive to physical crowding effects [14].

**FIGURE 1:**
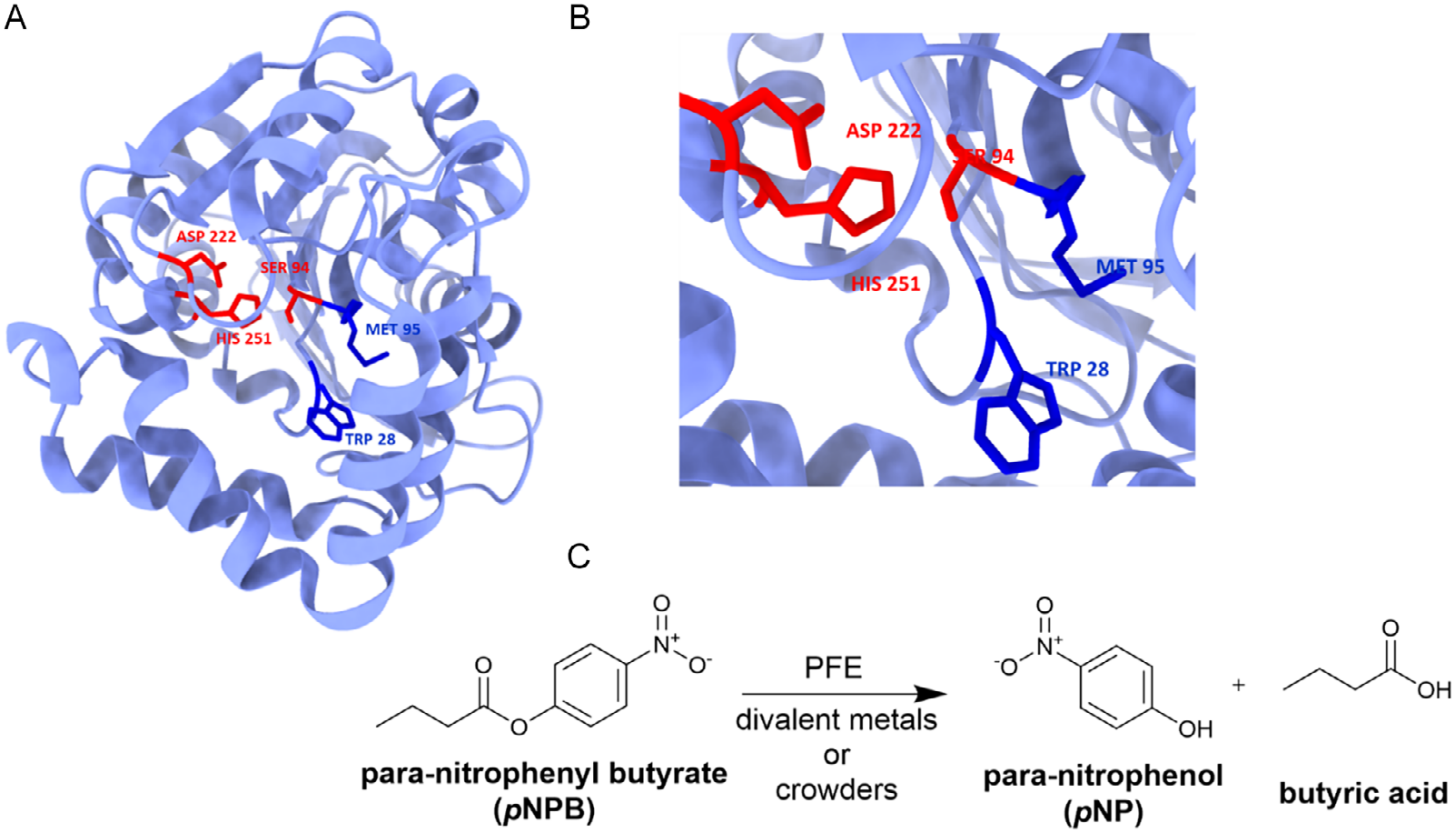
(A) X‐ray structure of *P. fluorescens* esterase I (PDB entry: 1VA4) [15]. The residues of the catalytic triad and of the oxyanion hole are shown as sticks in red and blue color, respectively. PFE was crystallized as a dimer of trimers; here, one monomer is shown for clarity. (B) As in (A), focusing on the catalytic triad and oxyanion hole residues. (C) Reaction catalyzed by PFE relevant to our study using *p*NPB as a model substrate.

A compelling model system to address such questions is the metal‐independent esterases from *Pseudomonas fluorescens*, specifically esterase I, PFE I (from herein PFE), a well‐studied enzyme (Figure 1A) [15]. PFE adopts the α/β‐hydrolase fold, featuring a buried active site (Ser94, His251 and Asp222) and a narrow tunnel formed by residues Trp28 and Met95, also mentioned as “oxyanion hole” residues (Figure 1B and Figure S1A) [16]. These structural features govern the substrate chain‐length specificity and enantioselectivity of PFE toward a variety of pharmaceutically relevant esters [17, 18]. PFE does not depend on a metal ion for its function, thereby serving as an ideal model system to disentangle specific metal effects from intrinsic catalysis. Structural engineering studies have shown that subtle reshaping of the active site and the oxyanion hole residues [19] can convert PFE from a short‐chain esterase to a catalyst for bulkier substrates or even to a promiscuous β‐lactamase [20]. Furthermore, focused directed evolution and OSCARR‐based cassette mutagenesis [21] have yielded PFE variants with increased catalytic efficiency and altered the enantioselectivity of synthetically valuable lactams and esters [22, 23]. These results demonstrate that even modest sequence changes can remodel the activity and selectivity of PFE, but they also require substantial efforts for molecular biology and high‐throughput screening [24, 25].

Here, PFE is employed as a model α/β‐hydrolase to investigate the effects of chemical and physical crowding on enzyme function. Specifically, we explore how a panel of divalent metal ions and micro‐, macromolecular crowders influence the biocatalytic activity and structural integrity of a mesophilic bacterial esterase. Building on prior structural and engineering studies on PFE, we systematically expose the enzyme to metal ions commonly encountered in industrial and environmental settings and to cell‐mimicking crowders polyethylene(glycol) (PEG), sucrose and glucose. Although metal ions and molecular crowding originate from different contexts, i.e. natural/industrial environments and intracellular conditions, respectively, they are physicochemical perturbators that can alter enzyme function and structure. We, therefore, address two key questions: (i) how a defined series of divalent cations perturbs PFE activity and solution behavior and (ii) whether molecular crowding alone suffices to alter enzyme performance. This study, by linking metal identity and crowding effects to changes in enzymatic activity, aims to provide a practical guide for the possibility of the deployment of PFE and related enzymes belonging to the large and ubiquitous α/β‐hydrolase family, in metal‐rich industrial environments and to highlight “physicochemical tuning” as a complementary strategy to protein engineering in industrial biocatalysis.

## 2 Results and Discussion

### 2.1 Metal‐Type and ‐Concentration Dependent Effects on PFE Activity

**FIGURE 2:**
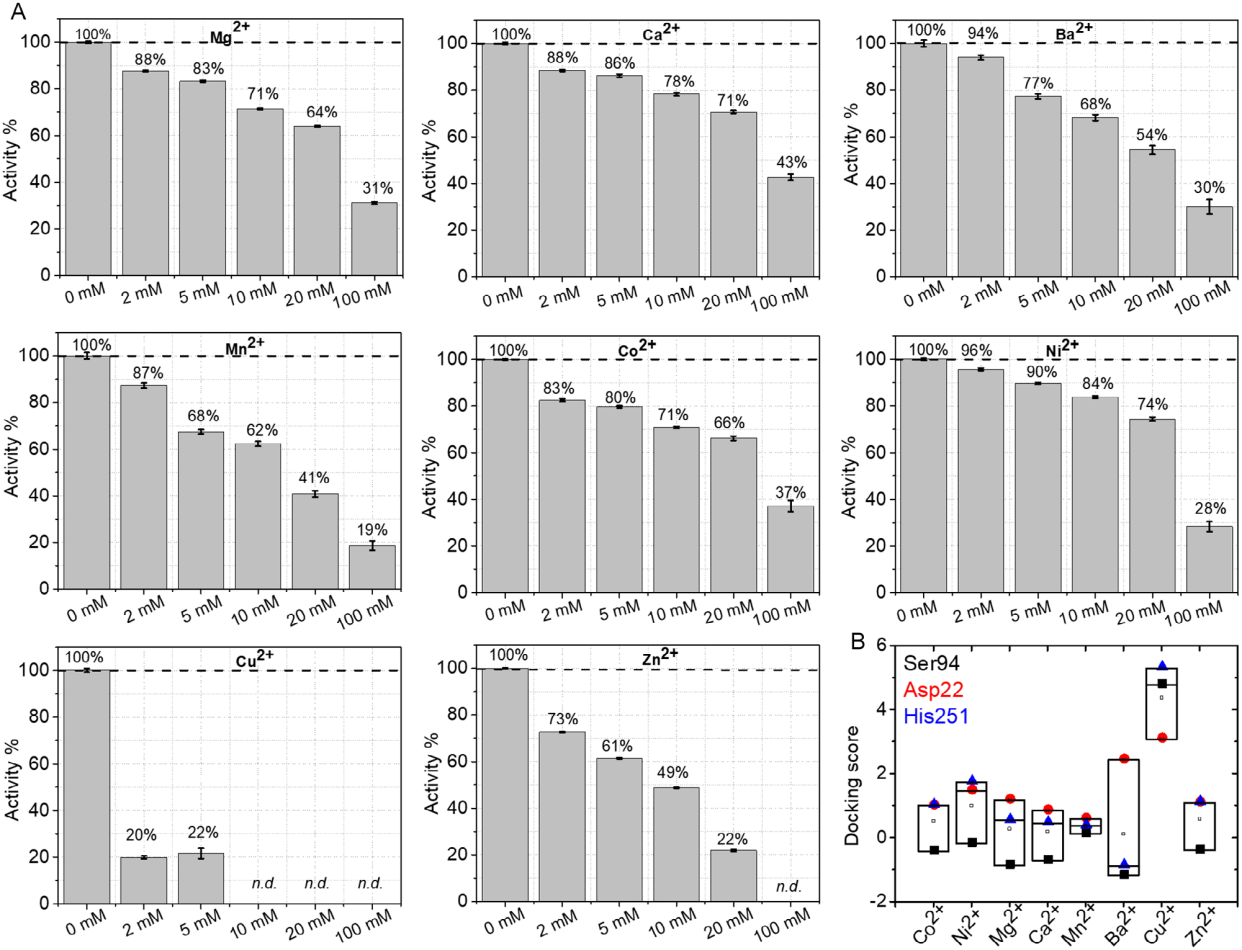
(A) Enzymatic activity of PFE (10 nM) using *p*NPB substrate (0.75 mM) at 27°C in absence and presence of increasing concentration of divalent metal ions (indicated); all metal ions were in the form of chlorides and error bars represent standard deviation from triplicates. Primary kinetic data along with linear fits are given in Figure S2A in Supporting Information; *n.d*. means not detected. Statistical analysis of 2 and 100 mM data versus native PFE is given in Table S1A, Supporting Information. Additional data on sulfate salts of Ni^2+^ and Zn^2+^ are given in Supporting Information, Figure S2B. (B) Qualitative in silico metal‐binding analysis of divalent metal ions toward PFE's active site using the MIB2 platform [28]; The *y* axis represents the normalized metal–residue interaction propensity score, and each point is a different residue of the active site (indicated).

PFE was expressed according to established protocols [22] and isolated in high purity as verified by sodium dodecyl sulfate‐polyacrylamide gel electrophoresis (SDS–PAGE) and mass spectrometry (Figure S1B,C). Under initial velocity conditions with *para*‐nitrophenyl butyrate (*p*NPB) as substrate (Figure 1C), the catalytic performance of PFE was monitored at 27°C in the absence and presence of various alkaline earth (Mg^2+^, Ca^2+^, and Ba^2+^) and transition (Mn^2+^, Co^2+^, Ni^2+^, Cu^2+^, and Zn^2+^) metal ions that are encountered in industrial and environmental settings, allowing a systematic comparison of metal‐dependent effects. *p*NPB was selected as model substrate due to its widespread use in esterase studies including investigation of metal effects, its minimal autohydrolysis, and straightforward photometric detection [26, 27]. Because the goal of this study is to assess enzyme robustness rather than a detailed catalytic mechanism, activity changes are reported as relative initial rates. At low metal concentrations (2 mM) relevant to industrial and ecological settings, which are yet five orders of magnitude higher than the enzyme concentration, PFE showed maintenance of its activity by ∼85%–95% for most metals (Figure 2A). Exceptions are Zn^2+^ and Cu^2+^, where activity dropped to ∼63%–73% (Figure S2B) and 20%, respectively. Statistical analysis confirmed significant differences between native and metal‐treated PFE for all metals at 2 mM (Table S1A). For descriptive purposes, metal‐induced inhibition was classified according to residual activity as significant (<50% residual activity), moderate (50%–80%) and weak (>80%). Thus, at 2 mM concentration, Cu^2+^ caused significant inhibition (∼20% residual activity), Zn^2+^ led to moderate inhibition, whereas Mg^2+^, Ca^2+^, Ba^2+^, Mn^2+^, Co^2+^, and Ni^2+^ caused weak variable inhibition; setting the latter metals compatible with catalysis under our experimental conditions, demonstrating PFE is resilient to them.

To better understand the effect of each metal ion on PFE's activity, experiments were performed at increasing metal concentrations (5, 10, 20, and 100 mM) under otherwise same experimental conditions (Figure 2A). While concentrations above typical industrial settings were included, these conditions were deliberately chosen to probe upper tolerance limits of PFE. For all metals, PFE exhibited statistically significant metal‐concentration dependent deactivation versus native PFE. Specifically, when PFE was monitored with increasing Cu^2+^ concentration, residual activity was only 20% for 5 mM metal (similar to 2 mM), and no product was detected at Cu^2+^ concentration >5 mM, suggesting complete enzyme deactivation. Following Cu^2+^, Zn^2+^ also exhibited complete deactivation with practically no residual activity at 100 mM metal. All other metals led to statistically significant variable inhibition (exhibiting ∼20%–40% residual activity) at 100 mM concentration. NiSO_4_^.^6H_2_O had only a weak effect on enzyme activity (Figure S2B), supporting counter ion effects. To quantitate the effect of each metal ion on PFE's activity, we estimated apparent IC_50_ values (Table S2 and Figure S2C). Cu^2+^ exhibited the strongest inhibitory effect, as expected (IC_50_ ∼ 0.4 mM), followed by Zn^2+^ (IC_50_ ∼ 2–10 mM). In contract, Mg^2+^, Ca^2+^, Ba^2+^, Mn^2+^, and Co^2+^ exhibited similar inhibitory effect (IC_50_ ∼ 12–15 mM), while Ni^2+^ had the weakest effect (IC_50_ ∼ 31 mM). The observed trends follow the Irving–Williams series [29] (Mn^2+^ < Fe^2+ ^< Co^2+^ < Ni^2+^ < Cu^2+^ > Zn^2+^) for the strongest binding metals, Cu^2+^ and Zn^2+^, however, deviations exist. Alkaline metals (Mg^2+^, Ca^2+^, and Ba^2+^) display inhibition comparable to some transition metals, and Ni^2+^ has a smaller inhibitory effect than expected. Overall, the concentration dependent deactivation observed for all metals suggests metal‐induced structural perturbation, and dedicated stability measurements would be required to confirm such effects.

To place our findings in the context of previous studies, we compared the effect of divalent metals on PFE with similar data on several lipases and esterases (Table S3). The comparison reveals several trends across systems: Cu^2+^ has the strongest inhibitory effect in nearly all cases, including PFE, which can be attributed to its high ligand affinity and strong propensity to interact with histidine and aspartate residues [27, 30, 31]. Zn^2+^ also causes enzyme deactivation, although with greater variability, also consistent with its strong coordination behavior that can block catalytic residues or promote crosslinking [32, 33]. By contrast, Ni^2+^ and Co^2+^ have more variable effects across systems, consistent with their intermediate position in the Irving–Williams series, suggesting enzyme‐specific factors like site accessibility and local coordination environment contribute to the observed effect. Finally, alkaline earth metals (Mg^2+^, Ca^2+^, and Ba^2+^) in general exhibit weak deactivation or have stabilizing effect (particularly for lipases), although exceptions exist. This behavior suggests that their effect stems from nonspecific electrostatic interactions, rather than direct interaction with catalytic residues, in agreement with their lower coordination strength.

Considering the above data and literature reports (Table S3), complete deactivation of PFE in the presence of already 10 mM Cu^2+^ could be attributed to possible direct interactions of Cu^2+^ ions with nitrogen‐ and oxygen‐containing residues such as His, Asp, and Ser, which are present in the catalytic triad of PFE. This behavior is consistent with its position in the Irving–Williams series [34, 35, 36, 37]. In addition, Cu^2+^ can adopt flexible coordination geometries due to Jahn–Teller distortion effects, allowing it to adapt more readily to the confined environment of the active site. To better understand copper behavior, complementary in silico docking analysis [28] was performed as a qualitative measure of metal‐binding propensity to the active site residues. The analysis indicated that binding is strongly metal‐dependent, with Cu^2+^ showing a higher predicted propensity to interact with Ser94, Asp222 and His251 (Figure 2B) providing a structural rationale for the experimentally observed Cu^2+^‐induced inhibition. However, the in silico approach does not constitute direct evidence of binding but indicates the likelihood of interactions. This is because factors such as pKa values, electrostatics and potential conformational dynamics are only implicitly captured by using patterns observed in known metal‐binding proteins, including residue preferences and typical coordination geometries [28]. In contrast, all other metals were found to be less potent to bind the catalytic site residues. Docking the metal ions to the oxyanion hole showed no significant interactions, suggesting that Trp28 and Met95 are unlikely to be directly involved in enzyme deactivation via metal coordination. Moreover, a broader docking analysis considering all possible residue–metal interactions (Figure S3 and Table S4 in Supporting Information) suggested that most metals preferentially interact with Asp residues away from the catalytic triad. This is consistent with the retention of activity for 10 mM Ni^2+^ (84%–90% depending on counter ion) and Co^2+^ (71%) samples, despite their known ability to coordinate His residues. Exception is again Cu^2+^, which showed a preference to interact with residues near (His218) or at (His251, Ser94) the active site, likely contributing to the observed loss of activity. Given that His‐Asp‐Ser catalytic triads are a defining feature of α/β‐hydrolase fold enzymes, similar sensitivity toward Cu^2+^ may be expected for related esterases with comparable active‐site accessibility.

### 2.2 Reversibility of Metal‐Induced Inhibition

**FIGURE 3:**
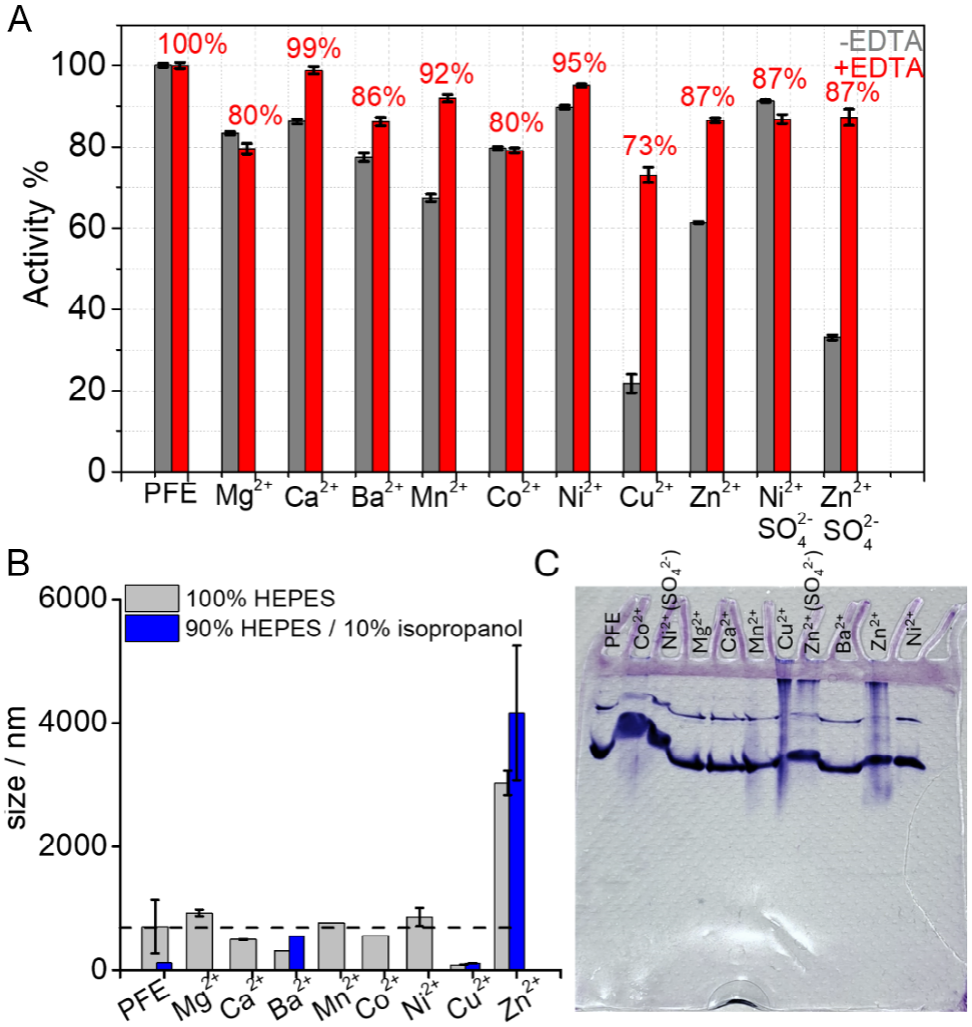
(A) Enzymatic activity of PFE (10 nM) using *p*NPB substrate (0.75 mM) at 27°C in the presence of 5 mM metal and in the absence (gray) or presence of 50 mM EDTA (red); error bars represent standard deviation from triplicates. Primary kinetic data along with linear fits are given in Figure S2A, S2B, and S2D. Statistical analysis of Figure 3A is given in Table S1B, Supporting Information. (B) DLS data of PFE (10 nM) in the presence of 20 mM metal ions at 25°C in HEPES (gray) and in HEPES/isopropanol 9/1 (blue) buffers. The horizontal dash marks the size of PFE alone in HEPES buffer; Ni^2+^ and Zn^2+^ were used in the form of sulfate salts, while all other metals in the form of chlorides; samples were measured once or twice (Zn^2+^ thrice), and error bars represent standard deviation from repeats. (C) Native–PAGE of PFE (17 μM in 10 mM HEPES buffer, pH 7.4) and of samples containing PFE in presence of 100 mM chloride salts. For Ni^2+^ and Zn^2+^ the sulfate salts were further assayed (indicated).

To evaluate whether metal binding and enzyme deactivation are reversible, the activity of PFE was measured in the presence of 5 mM metal and an excess of EDTA (50 mM) at 27°C (Figure 3A). Statistical analysis showed that PFE samples in presence of MgCl_2_, CoCl_2_, NiSO_4_ and treated with EDTA were not significantly different from their non‐EDTA treated counterparts, while for all other metals differences were significant (Table S1B), however to different degrees: Zn^2+^ (25%–54%) > Cu^2+^ (51%) > Mn^2+^ (24%) > Ca^2+^ (13%) > Ba^2+^ (9%) having final activity 87%, 73%, 92%, 99%, 86%, respectively. Comparable final residual activity (87%) was observed for both Zn^2+^ salts despite differences in starting inhibition levels. For descriptive purposes, the EDTA‐based recovery was classified according to the absolute increase in residual activity Δ_act_ = activity_(+EDTA) _– activity_(_–_EDTA)_, as follows: Δ_act_ ≤ 0% = no recovery, 0 < Δ_act_ < 10% = modest recovery, 10 ≤ Δ_act_ < 20% = moderate recovery and Δ_act_ ≥ 20% = significant recovery. Based on this classification, at 5 mM metal, PFE inhibition by Zn^2+^, Cu^2+^, and Mn^2+^ is significantly reversible, while by Ca^2+^ moderately reversible. All other metals are largely insensitive or have only a modest effect on the presence of excess EDTA in the mixture. Overall, for the two metals that showed the greatest inhibition (Cu^2+^ and Zn^2+^), EDTA recovered PFE's activity significantly suggesting the process is driven by reversible coordination or aggregation processes, rather than irreversible denaturation, with important implications in industrial feeds. Reversible inhibition by these two metals has previously been reported for other hydrolases [38, 39].

### 2.3 Metal‐Dependent Solution Behavior of PFE

To study whether metal‐induced aggregation is involved in PFE's deactivation, dynamic light scattering (DLS) measurements were performed in the presence of 20 mM metals at 25°C (Figure 3B, gray). Surprisingly, PFE alone had sizes of ∼500–600 nm. While self‐assembly of PFE is well‐known and manifested already at the X‐ray structure of the enzyme crystallized as dimer of trimers [15], with an effective volume of ∼212 nm^3^ corresponding to 7–8 nm diameter, there is a significant deviation from our experiment (7 vs. 500 nm). Such a discrepancy could be attributed to the inherent bias of the technique toward larger particles, which may overestimate the hydrodynamic radius (*D*_h_) in heterogeneous or partially aggregated systems [40]. Alternatively, the observed large size for native PFE may stem from self‐clustering of the enzyme due to its hydrophobic surface residues. The latter is further supported by the large polydispersity index (PDI) value of PFE (Figure S4). To test this hypothesis, DLS measurements were performed in presence of 10% isopropanol, expected to disrupt hydrophobic interactions. Indeed, in the presence of 10% isopropanol, PFE had a *D*_h_ of ∼130 nm (Figure 3B, blue) and less polydispersity (Figure S4). Even though isopropanol did not completely “dissolve” the clusters, the data support the notion that hydrophobic interactions are responsible for clustering of the native enzyme and the apparent large particle sizes. Addition of Mg^2+^, Ca^2+^, Ba^2+^, Mn^2+^, Co^2+^, and Ni^2+^ left this apparent size in the same order of magnitude (within experimental error) as native PFE (Figure 3B, gray), suggesting these ions do not significantly alter the apparent size distribution compared to native PFE. By contrast, Zn^2+^ shifted the apparent size to the micron range with the latter sample having *D*_h_ ∼ 4 μm, setting zinc as aggregation‐inducing metal, corroborating the strongest inhibition observed for Zn^2+^ compared to the other metals, Cu^2+^ aside (Figure 2B), as well as with the largest activity recovery observed with EDTA (Figure 3A). Addition of 10% isopropanol in the Zn^2+^‐loaded samples did not alter significantly the apparent size, suggesting Zn^2+^‐induced aggregation is not “destroyed” by disruption of hydrophobic interactions, in agreement with PDI values, which were further worsened (Figure S4). Interestingly, the Cu^2+^ sample had the smallest apparent size among samples (∼100 nm), both in the absence and presence of isopropanol, and smaller compared to native PFE.

To shed more light into the solution behavior of PFE in the presence of metals, we performed native gel electrophoresis (Figure 3C) that can provide information on enzyme oligomerization and higher‐order structures, as well as alterations of its conformation. Native PFE showed two distinct bands, suggesting the enzyme exists in two native forms; one (lower band) attributed to the monomeric form and the other (upper band) to an oligomeric state, in line with the DLS data. The addition of Mg^2+^, Ca^2+^, Ba^2+^, and Mn^2+^, did not alter the running profiles, suggesting no major conformational change or monomer↔oligomer equilibrium shift in the presence of these metals, again consistent with DLS observations. However, Co^2+^, Ni^2+^, Cu^2+^, and Zn^2+^ shifted the lower band, the latter reflecting changes in the net charge of the protein, which became less negative upon metal binding and thus affected electrophoretic mobility, or possibly reflecting a change in the oligomerization state of PFE in the presence of these metals. Furthermore, the shift of the band provides direct evidence of the binding of these metals to PFE. Interestingly, Cu^2+^ and Zn^2+^ bands further showed smearing and material stuck at the top of the gel. Both these features indicate sample heterogeneity and/or formation of aggregates consistent with DLS data for Zn^2+^, where aggregates were present. For Cu^2+^, the discrepancy may stem from the differences in the experimental conditions employed and the physical properties detected by the two techniques. DLS was done at the nM concentration regime, whereas native–PAGE at the low μM. Since aggregation and metal‐induced oligomerization are concentration‐dependent processes, the higher concentration used for native–PAGE could favor the formation of higher‐order species that are not equally present under DLS conditions. Moreover, native–PAGE is sensitive to changes in charge, conformation, and oligometic state, whereas DLS detects hydrodynamic sizes and may not detect compact or structural heterogeneous species with the same sensitivity as large aggregates. Finally, the extensive smearing could indicate enzyme degradation or misfolding; however, the partial restoration of activity for both metals upon addition of EDTA contradicts this scenario.

Overall, the inhibition trends qualitatively follow the Irving–Williams series only for the strongest‐binding transition metal ions, Cu^2+^ and Zn^2+^, which yield the strongest inhibitory effects. This means that metal binding affinities alone do not suffice to explain the trends observed in metal‐induced deactivation. Our combined data suggest that the outcome of metal binding depends on whether it promotes enzyme aggregation and whether it occurs at catalytically relevant residues. Native–PAGE provides qualitative evidence that Co^2+^, Ni^2+^, Cu^2+^, and Zn^2+^ associate with PFE as reflected by changes in their electrophoretic mobility. Furthermore, Zn^2+^ and Cu^2+^ promote the formation of aggregates consistent with their strong coordination properties and observed loss of activity. Zn^2+^‐induced aggregation has been observed in other proteins, including the amyloid β‐protein (Aβ) [41], β‐crystallins [33], creatine kinase [32], insulin [33], as well as hydrolases [42]; in the latter case, however, less explored and mechanistically understood. Accordingly, the pronounced aggregation observed for PFE in the presence of Zn^2+^, both with DLS and native–PAGE, agrees with trends of protein destabilization through zinc crosslinking and subsequent clustering. Similarly, Cu^2+^ is known to cause structural perturbation and formation of higher‐order species [43, 44] in line with our native–PAGE data on Cu^2+^‐treated PFE. Docking experiments also suggested Cu^2+^ binding at residues at or near the catalytic triad, contrary to all other metal ions, supporting catalytically disruptive binding in addition to aggregation. Ni^2+^ and Co^2+^ showed evidence of binding with comparatively weak inhibition, suggesting they are binding at less disruptive sites that do not strongly impair catalysis. Other metals have comparatively smaller effects on the size and structural integrity of PFE, indicating weaker, nonspecific interactions.

### 2.4 Metal‐Dependent Functional Stability Under Thermal Stress

**FIGURE 4:**
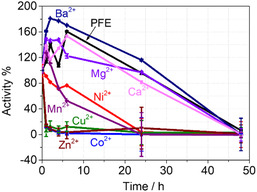
Metal‐dependent thermal deactivation of PFE (10 nM) incubated at 37°C in the presence of 5 mM divalent metal ions up to 48 h, with residual activity measured at 27°C using *p*NPB substrate (0.75 mM); error bars represent standard deviation from triplicates. Ni^2+^ and Zn^2+^ were used in the form of sulfate salts, while all other metals were used in the form of chlorides. Primary kinetic data, along with linear fits are given in Figure S2E.

To probe the effect of divalent metal ions on enzyme functional stability under mild thermal stress, PFE was preincubated at 37°C in the presence of 5 mM metal ions for up to 48 h, and residual activity was measured under standard conditions at predetermined time points at 27°C (Figure 4). Representative % activity values at 2, 4, and 24 h are given in Table S5. PFE, Mg^2+^‐, Ca^2+^‐, and Ba^2+^‐treated PFE exhibited enhanced activity during early incubation times, suggesting transient stabilization or altered conformational dynamics under mild thermal stress [17]; consistent with weak, nonspecific interactions of these metals with the protein. Particularly, Ca^2+^‐ and Ba^2+^‐loaded PFE had a ∼80% and ∼60% maximal increase at early times, similar to native PFE (∼60%). Mn^2+^ and Ni^2+^ showed intermediate loss of stability, while Cu^2+^ and Zn^2+^ led to rapid and near‐complete loss (∼90%) of residual activity within 1 h. The thermal stress data on Cu^2+^ and Zn^2+^ corroborate with the largest inhibition observed for these metals at 27°C without heat treatment (Figure 2A), as well as with the largest recovery in activity upon treatment with EDTA (Figure 3A), suggesting that metals inducing strong inhibition at 27°C also promote functional destabilization under thermal stress. Interestingly, Co^2+^ also induced strong deactivation under thermal stress, despite showing only moderate inhibition at 27°C, suggesting a distinct mechanism related to reduced structural stability rather than direct interference with catalytic residues, in line with native–PAGE observations. In contrast, Mg^2+^, Ca^2+^, and Ba^2+^ samples that showed similar weak‐to‐moderate deactivation (Figure 2A) exhibited early time activation under thermal stress. The thermal stress experiments broadly follow the expected trend of Irving–Williams series, with alkaline metals showing activation of PFE at early incubation times, Mn^2+^ moderate inhibition and Cu^2+^, Zn^2+^ significant deactivation. Notably, Ni^2+^ exhibited a moderate inhibition under these conditions. Because nickel sulfate was employed in this experiment, the contribution from counterion effects cannot be excluded. The discrepancy on Co^2+^ suggests that metal effects on PFE are condition‐dependent, with Co^2+^ exerting a stronger impact on thermal stability than on catalytic activity under thermal stress. Nonetheless, prolonged incubation led to progressive deactivation for all samples, including native PFE.

### 2.5 PFE Activity Under Molecular Crowding Conditions

**FIGURE 5:**
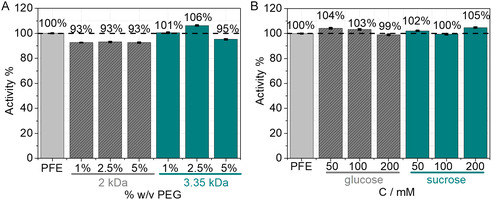
Enzymatic activity of PFE (10 nM) using *p*NPB substrate (0.75 mM) at 27°C in the presence of increasing concentration (A) macro‐ and (B) micromolecular crowding; In (A), activity in the presence of 2 and 3.35 kDa PEG is shown in dark gray and cyan, respectively, whereas in (B) activity in presence of glucose and sucrose is shown in dark gray and cyan, respectively versus native PFE (in light gray); error bars represent standard deviation from triplicates. Primary kinetic data, along with linear fits, are given in Figure S2F.

Aside from chemical crowding induced by metals, PFE's robustness was further tested against physical crowding. Specifically, the catalytic activity was measured at 27°C in presence of macro‐ (PEG 2.0 and 3.35 kDa at 1, 2.5, and 5% w/v) and micro‐ (sucrose and glucose at 50, 100, and 200 mM) molecular crowders (Figure 5). Little to no effect was observed on PFE's activity across the PEG type and concentration range tested (1%–5% w/v) with residual activities staying withing ∼ 5%–10% of the PEG‐free controls (Figure 5A). Likewise, the addition of small molecule crowders at 50–200 mM did not substantially alter the enzymatic performance, and catalytic activity was close to 100% (Figure 5B). A slight enhancement of ∼5% was observed for some of the highest concentrations tested; sucrose 200 mM and PEG 3.35 kDa 2.5% w/v. To estimate whether viscosity was significantly altered in our solutions, we performed viscosity measurements and compared them to a sample containing only buffer (Table 1). We found that PEG increased the viscosity of the solution substantially (40%–60%) compared to buffer, while sugars only moderately (10%–15%). Despite this, the activity of PFE was preserved in all samples, suggesting PFE's activity is not impaired by increased viscosity and excluded‐volume effects [45]. One might anticipate that the crowders may slow down the diffusion of the substrate (*p*NPB) and of the product (*p*NP) [45]; however, catalysis was not diffusion‐limited or osmolyte‐sensitive under our experimental conditions. Additionally, the data suggests that molecular crowders do not alter enzyme conformation [46] and active site geometry, block the catalytic site or compete with the binding of the substrate. The latter is particularly relevant for glucose that has a molecular size of 161 Å^2^, which lies between the molecular sizes of the *p*NPB (203 Å^2^) and (*p*NP) (126 Å^2^). It should also be pointed out that the densities of the above solutions increased only marginally (<2%) compared to buffer (Table 1) suggesting any changes in their volumes are negligible. Overall, PFE exhibited resilience to macro‐ and micromolecular crowding, yielding residual activities within 5%–10% of controls and modest ∼5% enhancement at high concentrations of PEG 2.5% and sucrose 200 mM, suggesting stabilization via hydrophobic or viscosity effect. This tolerance aligns with PEG's suppression of enzyme aggregation [47] and sucrose‐induced helical stabilization [48], contrasting sensitivity in less robust hydrolases like NS3/A4 [49]. Overall, our data in the presence of crowders highlight the catalytic robustness, and by extension, structural integrity, of PFE under conditions that mimic the crowded cellular environments.

**TABLE 1:** Viscosity and density of selected crowding conditions at 25°C.

Conditions	Viscosity, mPa.s	**Density, g/cm** ^**3**^
Buffer	0.9201	1.0050
PEG 2 kDa 5% w/v	1.2819	1.0112
PEG 3.35 kDa 5% w/v	1.4832	1.0115
Glucose 200 mM	1.0016	1.0173
Sucrose 200 mM	1.0551	1.0247

## 3 Conclusions

Using PFE as a representative α/β‐hydrolase, we investigated enzyme performance in presence of chemical (metal ions) and physical (molecular crowding) perturbations. PFE exhibited catalytic tolerance against a series of divalent metal ions at 2 mM concentrations relevant to industrial and ecological settings and a pronounced dependence on divalent metal ion identity and concentration. Specifically, Cu^2+^ deactivated PFE at concentrations higher than 5 mM, followed by Zn^2+^, whereas the presence of other divalent metal ions had weak to moderate effects under these conditions. By increasing metal concentrations to regimes above industrial and environmental settings, PFE showed progressive deactivation. The data suggest that at high metal concentrations, the structural integrity of PFE is compromised, requiring an understanding of its underlying stability and structural integrity under these conditions.

Collectively, experiments on EDTA recovery, in silico docking, DLS, native–PAGE and activity studies under thermal stress, reveal that metal‐dependent effects arise from distinct mechanisms. Zn^2+^ and Cu^2+^ induced the strongest deactivation, via aggregation/structural heterogeneity, consistent with literature reports. Docking studies further suggest Cu^2+^ interacts with residues at or near the catalytic triad, but this is yet to be confirmed experimentally. By contrast, Ni^2+^ and Co^2+^ exert weaker effects on PFE's activity and structural integrity, demonstrating that metal binding alone is not sufficient to predict the strength of inhibition and emphasizes metal coordination is not necessarily coupled to inhibition. Interestingly, the phenomenon of metal‐induced enzyme aggregation as observed for Zn^2+^ and Cu^2+^ can be exploited in biocatalysis as an alternative immobilization approach, through the formation of so‐called nanoflower structures, often yielding enhanced catalytic activity, enzyme stability, as well as improved storage time [50, 51].

The pronounced inhibitory behavior of Cu^2+^ and Zn^2+^ agrees qualitatively with literature reports in esterases and hydrolases (see Table S3). However, our findings further suggest that inhibition strength is dependent not only on metal affinities but also on the structural consequences of metal coordination, including aggregation and active‐site coordination. Given that the His‐Asp‐Ser catalytic triad is present in α/β‐hydrolases, susceptibility to Cu^2+^ may extend to related enzymes, though inhibition is expected to depend on other factors like active‐site accessibility and aggregation propensity. These factors may also explain why PFE does not strictly follow inhibition trends predicted from the Irving‐Williams series or previous esterase comparisons.

In contrast to chemical perturbation, PFE retained catalytic competence under both macro‐ and micromolecular crowding (PEG 1%–5% w/v; glucose and sucrose 50–200 mM), demonstrating that high viscosity and excluded volume effects do not perturb its activity and conformation. The resilience of PFE at both moderately metal‐loaded and crowded conditions at mesophilic temperatures (27°C) supports its deployment in sustainable biocatalytic processes, such as enantioselective hydrolysis in metal‐laden or viscous feeds.

Future work should focus on (i) using variable substrates to identify potential substrate‐dependent enzyme behavior and assess the generality of the observed effects, (ii) investigating structural stability under metal‐loaded conditions, and (iii) exploring immobilization strategies to exploit these traits at scale.

## 4 Experimental

### 4.1 Enzyme Production and Purification

The recombinant *P. fluorescens* esterase I was produced in *E. coli* strain BL21(DE3) harboring the pGASTON plasmid carrying the gene encoding PFE I. Overexpression was performed in 1 L LB medium containing 50 μg/μL ampicillin. Cultures were incubated at 37°C, 90 rpm until OD_600_ reached 0.5. Then, induction was done with isopropyl‐β‐D‐thiogalactoside (IPTG) at 0.2 mM final concentration, followed by incubation at 37°C for 5 h or at 18°C for 16 h. Cells were harvested by centrifugation at 16,500 × *g* for 30 min and at 4°C, and the cell pellet was either lysed immediately or stored at −20°C.

Protein purification was performed in a cold room (∼7°C). Cells were resuspended in 50 mM HEPES pH 7.5, 300 mM NaCl, 5 mM imidazole, and lysed with sonication (amplitude 50%, 2.5 min on/2.5 min off, for a duration of 25 min) using a sonication probe (Branson 250 sonifier). The lysate was centrifuged at 6000 × *g* for 40 min at 4°C, and the supernatant was loaded to a chromatography column of 1.5 cm diameter loaded with Ni‐NTA resin. The column was washed with 3 mL × 3 wash buffer A (20 mM imidazole) and 3 mL × 3 wash buffer B (100 mM imidazole) in 50 mM HEPES pH 7.5, 300 mM NaCl to remove nonspecifically bound proteins. PFE was eluted with elution buffer (300 mM imidazole, 50 mM HEPES, pH 7.5, 300 mM NaCl) and fractions containing the enzyme were pooled, concentrated by Vivaspin concentrator 10 kDa cutoff, and buffer‐exchanged into storage buffer (10 mM HEPES, pH 7.5). Protein concentration was determined by the Bradford assay and absorbance at 280 nm (*A*_280_), with both methods giving convergent results.

### 4.2 Enzyme Quality Control (SDS–PAGE and MALDI–TOF Mass Spectrometry)

Enzyme purity was assessed with SDS–PAGE and MALDI–TOF spectrometry. For SDS–PAGE, samples (16 μL) were mixed with sample buffer 5× (5 μL) and were heated to 95°C for 5 min before being loaded to the gel (composition: 7% and 12% stacking and resolving gels, respectively). Samples were run at 160 V for 1 h, and the gels were stained with Coomassie brilliant blue G‐250 followed by destaining.

For MALDI–TOF, 2 μL of purified PFE were mixed with 2 μL matrix and mounted to the MTP AnchorChip. Synapinic acid (SA) was used as matrix in 50% acetonitrile/50% water (v/v) with 0.1% TFA (v/v), at 10 μg/μL. MALDI–TOF was run in linear positive mode to check the enzyme's purity based on the theoretical molecular weight of PFE (31,059 Da).

### 4.3 Enzymatic Activity Assay

The activity of PFE was evaluated at 27°C using substrate *p*NPB by monitoring the absorbance of the product *para*‐nitrophenol (*p*NP) (Figure 1C) at 405 nm for ∼15 min using a 96‐well plate and a multiscan plate reader (ThermoFischer Scientific). All measurements were conducted in triplicates. *p*NPB was prepared as a 10 mM stock solution in DMSO for complete solubilization. The final reaction mixture contained 10 nM PFE in 10 mM HEPES buffer, pH 7.5, and 0.75 mM *p*NPB at a 200 μL final reaction volume. A low enzyme concentration was mandated to ensure enzyme kinetic measurements under linear initial velocity conditions without saturation of the UV spectrometer receiver. Accordingly, the study focuses on relative initial rates as a practical metric of catalytic robustness under perturbing conditions rather than full Michaelis–Menten characterization. The optimal pH for esterase activity using a similar substrate (*para*‐nitrophenol acetate, *p*NPA) is 7–9 [52]; here, a pH 7.5 was used to minimize autohydrolysis of *p*NPB, facilitated at higher pH values and compromising result accuracy. The extinction coefficient (*ε*) of *p*NP under our experimental conditions was found to be 9956 mM^−1 ^cm^−1^.

For evaluating the effect of metal ions on PFE activity, stock solutions (1 M and 500 mM in distilled H_2_O) were prepared from sulfate and chloride salts (NiCl_2_, NiSO_4_ · 6H_2_O, CoCl_2_ · 6H_2_O, MgCl_2_ · 6H_2_O, CaCl_2_ · 2H_2_O, ZnCl_2_, ZnSO_4_ · 7H_2_O, BaCl_2_·2H_2_O, MnCl_2_ · 4H_2_O, and CuCl_2_·2H_2_O) and added at increasing concentrations to yield final metal concentrations 2, 5, 10, 20, and 100 mM, whereas PFE and *p*NPB were kept constant at 10 nM and 0.75 mM, respectively. Control experiments (substrate + metal, no PFE) were performed to assess potential metal‐dependent contributions to absorbance. At 10 mM metal concentration, only ZnSO_4_ · 7H_2_O and CuCl_2_·2H_2_O samples gave measurable background absorbance (Figure S2G), while all other metals showed no significant effects. Accordingly, for these two metals, controls containing *p*NPB + metal were included at each concentration and subtracted from the corresponding activity data prior to data fitting. For all other metals, a single control (0.75 mM *p*NPB + 10 mM metal) was used for all the metal concentrations tested.

For monitoring the effect of crowders, PEG (2 kDa and 3.35 kDa; 50% w/v stocks in buffer) was added to 1%, 2.5%, and 5% w/v final concentrations, while glucose and sucrose (2 M stocks in buffer) were added to 50, 100, and 200 mM final concentrations. In all enzymatic activity studies, the buffer (10 mM HEPES, pH 7.5) volume was adjusted according to additive concentration to ensure a constant 200 μL final volume and as control, a sample containing 0.75 mM *p*NPB + crowder (at the concentration used for the assay) in buffer was used.

For assessing the reversibility of metal binding to PFE, activity assays were performed at 10 nM PFE in the presence of 5 mM metal ions and 50 mM EDTA. EDTA stock solutions (250 mM) were prepared in distilled H_2_O, and the pH was adjusted to 8.0 using 4 M NaOH prior to being added to the reaction well. Following the addition of EDTA to the PFE + metal mixture, samples were incubated for 20 min at room temperature to ensure quantitative chelation by the EDTA. After this time, the reaction was initiated by addition of *p*NPB (at final concentration of 0.75 mM) and the absorbance was monitored as described above. Control experiments (0.75 mM *p*NPB + 5 mM metal + 50 mM EDTA, no enzyme) were performed under identical buffer conditions as in the activity assay in the presence of the enzyme.

For thermal stress experiments, 10 nM PFE samples were incubated at 37°C in the presence of 0.75 mM *p*NPB and 5 mM metal ions for up to 48 h. At selected time points, aliquots of predetermined volume were removed, and their catalytic activity was measured at 27°C as described above. Control experiments (0.75 mM *p*NPB + 5 mM metal, no enzyme) were performed at the initial time point, and the corresponding background signal was subtracted from all subsequent measurements for each metal.

All kinetic data (*A*_405_ vs. time) were analyzed in OriginPro 8.5 or 9 by fitting the data to a linear function to estimate the initial rate (slope). For all experiments the slope of PFE alone under experimentally identical conditions was used as control corresponding to 100% activity and activity across different conditions was expressed relative to the control.

### 4.4 DLS Measurements

Samples for DLS were prepared at 10 nM PFE in 10 mM HEPES buffer, pH 7.5, followed by filtering using a 0.2 μm Whatman syringe filter. Hydrodynamic size measurements were performed using a Zetasizer Nano ZS90 (Malvern Paranalytical Ltd.) at 25°C, where size distributions were fitted automatically, and the error bars represent the error associated with different measurements, with most samples measured twice (Zn^2+^ thrice). PDI values of Figure 3B are given in Supporting Information, Figure S4.

### 4.5 Native–PAGE Analysis

PFE samples (17 μM in 10 mM HEPES buffer, pH 7.5) in the absence and presence of 100 mM metals were analyzed by native–PAGE (composition: 4% and 10% stacking and resolving gels, respectively) under nondenaturing conditions. Samples (15 μL) were mixed with native loading buffer 5× containing glycerol (5 μL) and were loaded without being heated. Samples were run at 160 V for 1 h, and the gels were stained with Coomassie brilliant blue G‐250 followed by overnight destaining. A higher protein concentration was used here compared to the rest of the experiments (10 nM) to allow visualization of the bands on the gel.

### 4.6 Density and Viscosity Measurements

Density and viscosity were measured at 25°C using an Anton Paar DMA 4100 M instrument equipped with a Lovis rolling‐ball viscometer. Samples (4 mL each) were equilibrated at 25°C, and viscosity was determined from rolling‐ball transit times. Density was measured using an oscillating U‐tube.

### 4.7 In Silico Metal Docking

In silico metal docking was performed using the X‐ray structure of PFE (PDB entry: 1VA4) [15] in its monomeric form without ligands and the online platform MIB2 [28] using default parameters to assess the binding affinity of each metal to the catalytic triad residues (Figure 2B) or to all PFE residues (Figure S3 and Table S4). This tool predicts metal‐binding sites based on structural templates derived from experimentally characterized metal‐binding proteins, taking into account residue identity, coordination geometry, and local structural environment. The resulting scores reflect the likelihood of metal–residue interactions, with higher values indicating stronger predicted interactions and can be therefore be used comparatively within the same protein system rather than as an absolute thermodynamic binding energy.

### 4.8 Estimation of IC_50_ Values

The residual activity data versus metal concentration were fitted (Figure S2C, Supporting Information) in OriginPro 9 to a monoexponential decay function of the form $y=A_{1}e^{-\frac{x}{t_{1}}}+y_{0}$, where y is the % residual activity, x the metal concentration (in mM), $A_{1}$ the decay amplitude, $y_{0}$ the plateau of residual activity reached and $t_{1}$ the decay constant. As apparent IC_50_ was defined as the metal concentration causing 50% loss of initial activity estimated from $IC_{50}=t_{1}ln2$ (Table S2, Supporting Information). For NiSO_4_^.^6H_2_O which did not affect residual activity >90%, the IC_50_ value was not determined.

### 4.9 Statistical Analysis of Catalysis Data

Statistical analysis of enzymatic activity data (Figure 2A and 3A and Figure S2B) was performed with OriginPro 9 by one‐way ANOVA followed by Holm‐Bonferroni correction. For Figures 2A and S2B, differences between native versus metal‐treated PFE were assessed for 2 and 10 mM metal concentrations using triplicate measurements with the results summarized in Table S1A. For Figure 3A statistical analysis was performed to assess the significance of EDTA‐based recovery of activity for a given metal (at 5 mM) using triplicate measurements with the results summarized in Table S1B. Statistical analysis is denoted as follows: ns (not significant, *p* ≥ 0.05), ** (*p* < 0.01), and *** (*p* < 0.001). For Figure 3A the data were further classified based on Δ_act_ = activity_(+EDTA)_ – activity_(_–_EDTA)_ as described in the text.

## Conflicts of Interest

The authors declare no conflicts of interest.

## Supporting information

Supplementary Material

## Data Availability

The data that supports the findings of this study are available in the supplementary material of this article.
