# Supplementary material for: Metal‐Dependent Effects and Crowding Robustness of Pseudomonas fluorescens Esterase I
Source: Chembiochem. 2026 Jun 7;27(11):e70424. doi: 10.1002/cbic.70424 (PMC13244118; doi:10.1002/cbic.70424)
Supplement: Supplementary file 1 — Supplementary Material [file CBIC-27-e70424-s001.pdf]

Supporting Information

Metal-Dependent Effects and Crowding Robustness of *Pseudomonas fluorescens* Esterase I

Emmanouil Ntermanakis,[a],^ Nikoleta Syngelaki,[a],^ Alexandros Lyratzakis,[a] Renia Fotiadou,[a] Spyridoula Charova,[a] Ioannis V. Pavlidis[a] and Angeliki Giannouli [a],*

[a]Department of Chemistry, University of Crete, 70013 Heraklion, Greece
*Corresponding author: agiannouli@uoc.gr
^ these authors contributed equally

Table of Contents

1. Sequence and characterization of PFE S2
2. Additional and primary catalysis data S3
3. Additional *in silico* analysis S7
4. Additional solution behavior data S8
5. Tables S8
6. References S10

1. **Sequence and characterization of PFE**

Sequence

MSTFVAKDGT QIYFKDWGSG KPVLFSHGWL LDADMWEYQM EYLSSRGYRT IAFDRRGFGR SDQPWTGNDY DTFADDIAQL IEHLDLKEVT LVGFSMGGGD VARYIARHGS ARVAGLVLLG AVTPLFGQKP DYPQGVPLDV FARFKTELLK DRAQFISDFN APFYGINKGQ VVSQGVQTQT LQIALLASLK ATVDCVTAFA ETDFRPDMAK IDVPTLVIHG DGDQIVPFET TGKVAAELIK GAELKVYKDA PHGFAVTHAQ QLNEDLLAFL KRGSHHHHHH

Characterization

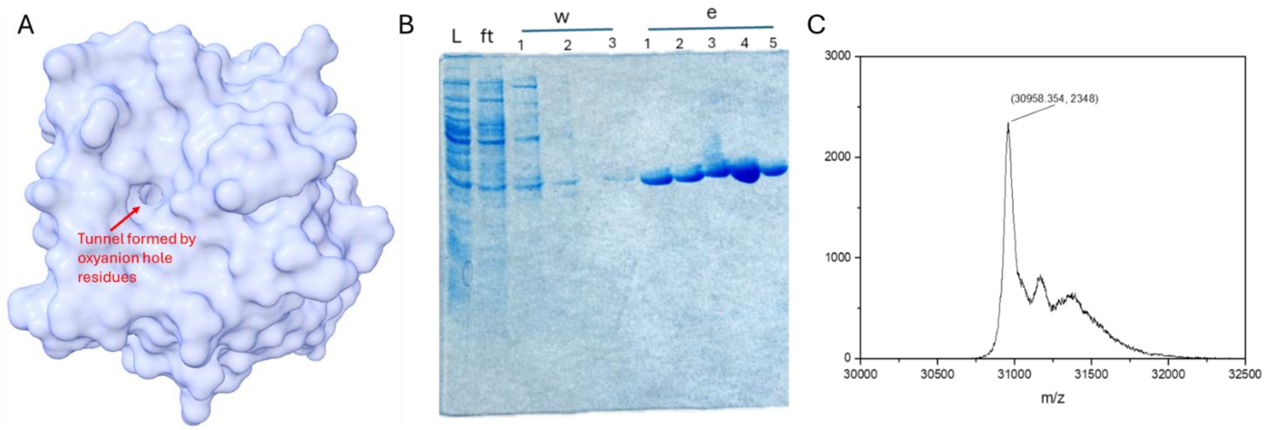

Figure S1. A) Surface representation of PFE with the narrow tunnel indicated. B) SDS-PAGE after Ni^{2+} affinity column chromatography showing PFE at the expected molecular weight (MW) of ~31 kDa (L: cell lysate, ft: flow through, w: wash, e: elution); conditions: 12% acrylamide, 160 V. C) MALDI–TOF spectrum of purified PFE (15 μM) using sinapinic acid matrix. The observed main peak (30,958 Da) is ~101 Da lower than the expected MW (31,059 Da) which could be attributed to a combination of amino acid cleavage and metal adduct.

2. Additional and primary catalysis data

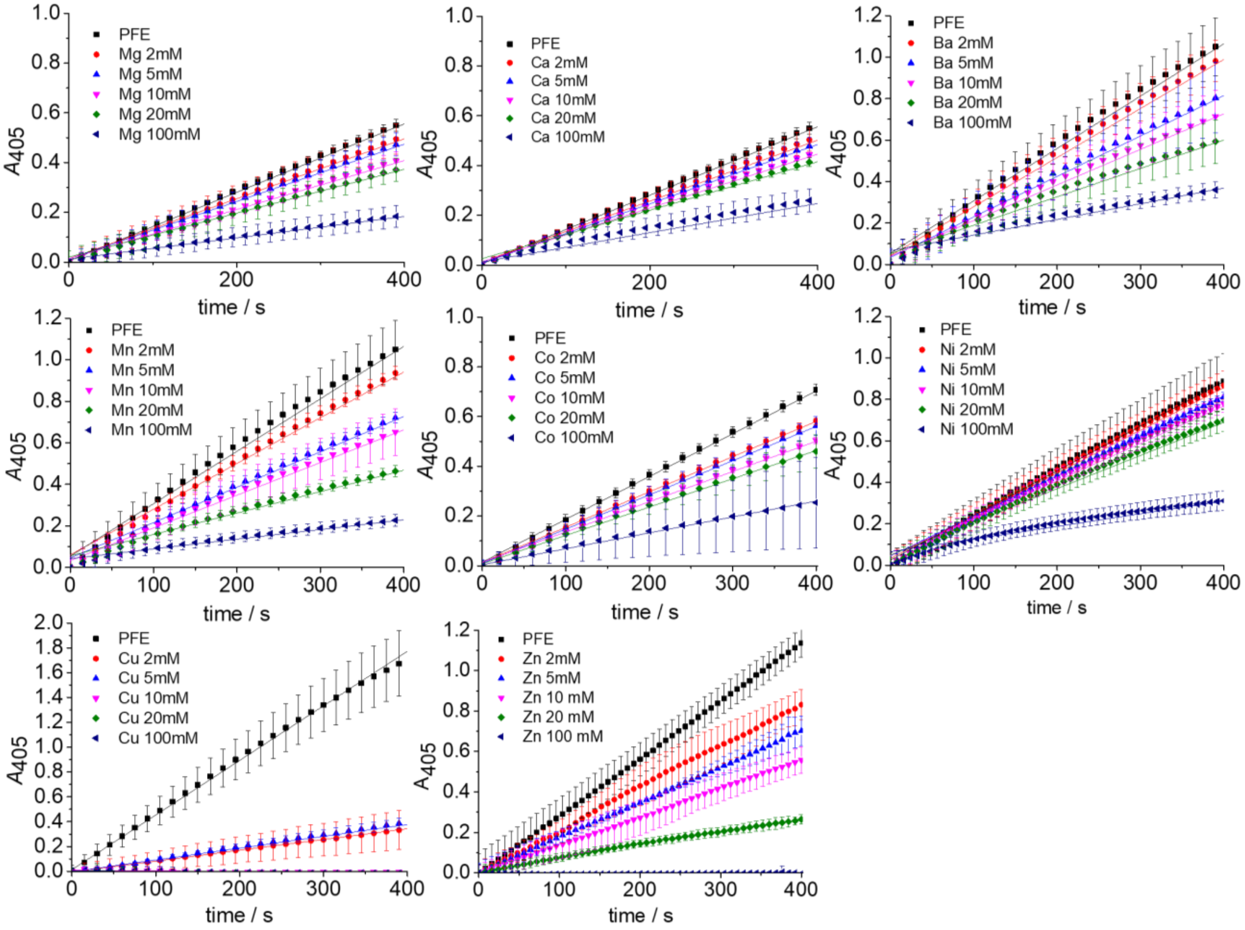

Figure S2A. Primary kinetic data of **Figure 2A**; Catalytic activity of PFE (10 nM) in presence of increasing metal concentrations (indicated) using *p*NPB (0.75 mM) as substrate at 27°C.

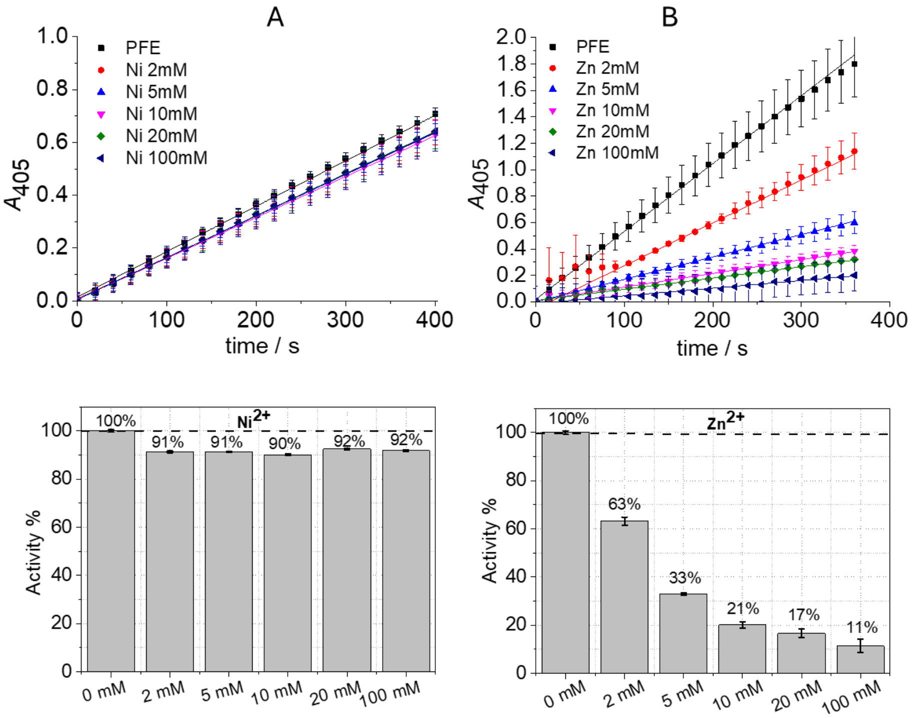

Figure S2B. Primary kinetic data (top) and % enzymatic activity of PFE (10 nM) using *p*NPB substrate (0.75 mM) at 27°C in absence and presence of increasing concentration of (A) $NiSO_4 \cdot 6H_2O$ and (B) $ZnSO_4 \cdot 7H_2O$; error bars represent standard deviation from triplicates.

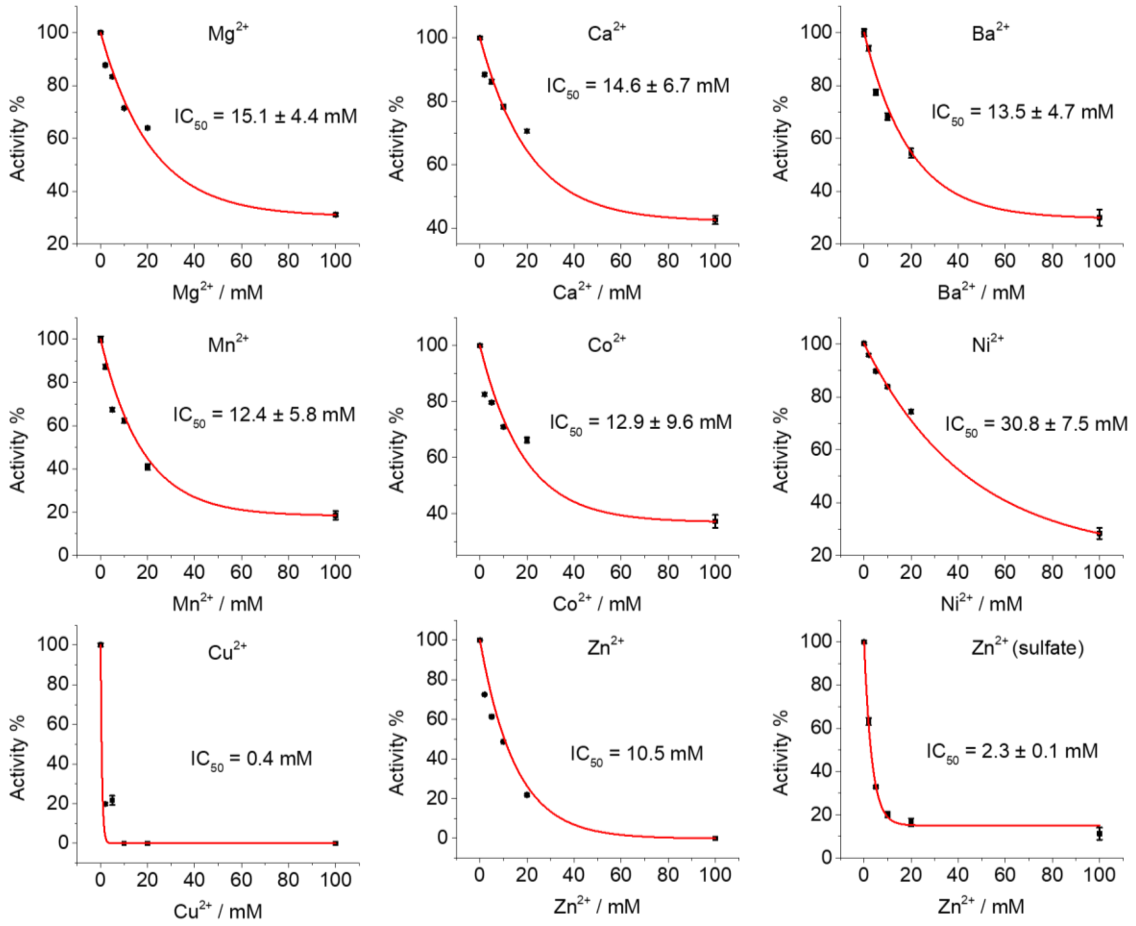

Figure S2C. Residual activity of PFE in presence of increasing concentration of divalent metal ions (indicated). Data were fitted using a mono-exponential decay function (red line) from which t_1 was derived (see **Table S2**). The IC_{50} values were stimated using $IC_{50} = t_1 ln2$.

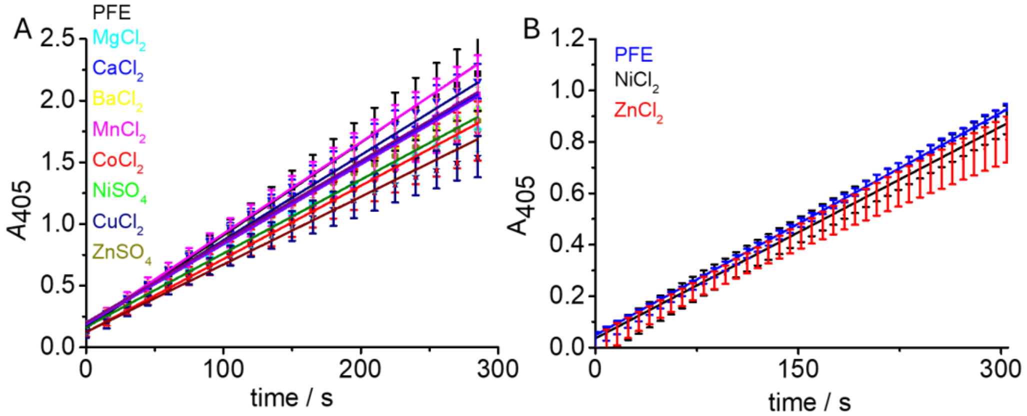

Figure S2D. Primary kinetic data of **Figure 3A**; Catalytic activity of PFE (10 nM) in presence of 5 mM metal (indicated) and excess of EDTA (50 mM) using *p*NPB (0.75 mM) as substrate at 27°C. Corresponding primary data in absence of EDTA are given in **Figure S2A** and **S2B**.

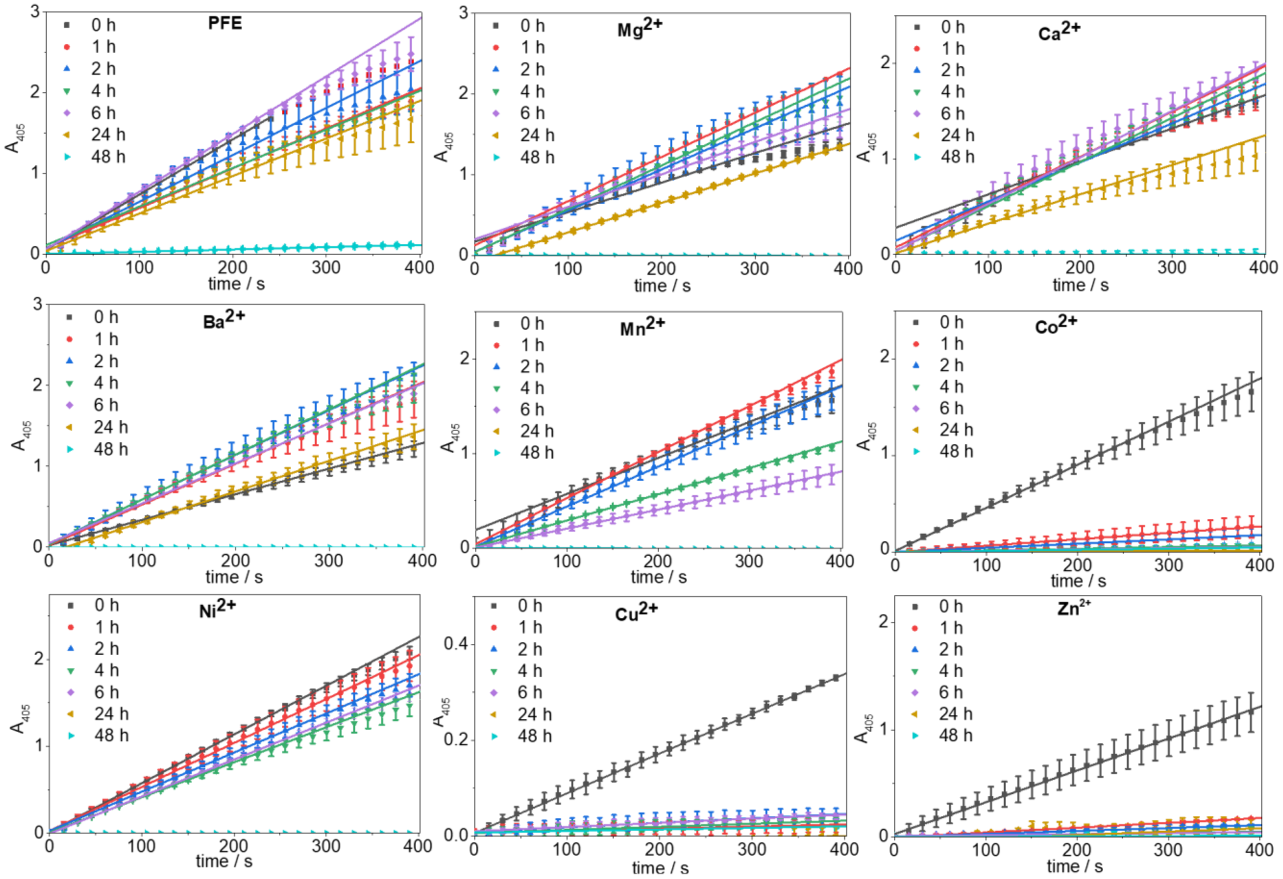

Figure S2E. Primary kinetic data of **Figure 4**; Catalytic activity of PFE (10 nM) in presence of 5 mM metal (indicated) using *p*NPB (0.75 mM) as substrate at 27°C at different times (indicated) upon incubation at 37°C.

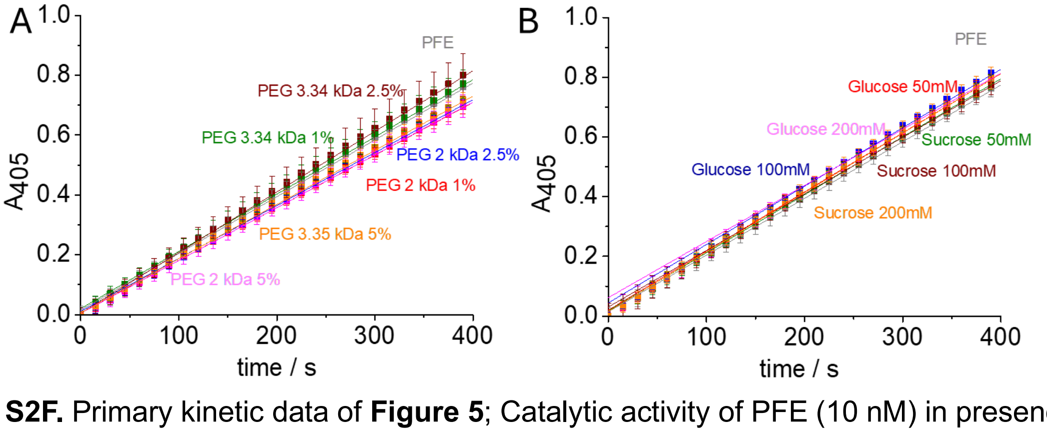

Figure S2F. Primary kinetic data of **Figure 5**; Catalytic activity of PFE (10 nM) in presence of (A) macromolecular crowders PEG 2, 3.35 kDa and (B) micromolecular crowders glucose, sucrose using *p*NPB (0.75 mM) as substrate at 27°C.

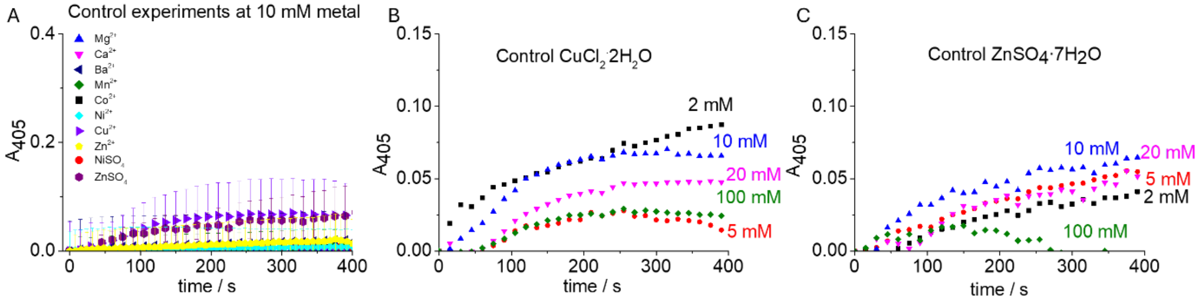

Figure S2G. Primary kinetic data of control experiments at 27°C containing 0.75 mM *p*NPB, no enzyme and (A) 10 mM metal, used to assess metal-dependent absorbance effects, (B) increasing concentrations of (B) $CuCl_2{\cdot}2H_2O$ and (C) $ZnSO_4{\cdot}7H_2O$. In (A) all metals are in the form of chloride salts, while Ni^{2+} and Zn^{2+} were further assayed in their sulfate form (indicated). The data in (B, C) served as control and were subtracted from corresponding experiments in the presence of PFE prior to data fitting to a linear function.

3. Additional *in silico* analysis

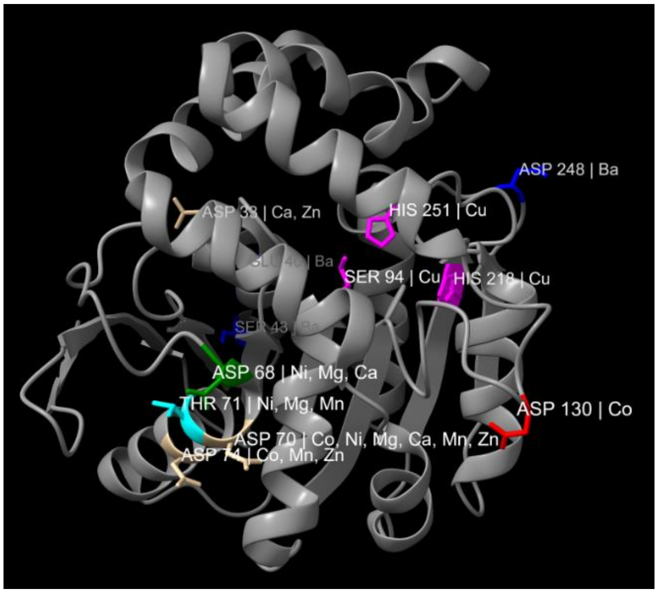

Figure S3. Structure of PFE showing all the residues that have high propensity to bind the divalent metal ions used in the study using the online platform MIB2 and default parameters. The results are also summarized in **Table S4**.

4. Additional solution behavior data

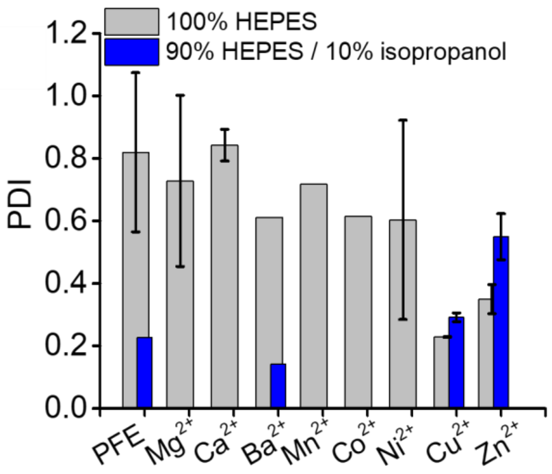

Figure S4. PDI values corresponding to measurements shown in **Figure 3B**.

5. Tables

Table S1A. Holm-Bonferroni statistical analysis of **Figure 2A** and **S2B** for 2 and 10 mM metal concentration between metal-treated PFE and native PFE. Statistical significance is denoted as ns (not significant, p ≥0.05), ** (p<0.01) and *** (p<0.001).

metal	2 mM metal + PFE vs. PFE	100 mM metal + PFE vs. PFE
$MgCl_2$	***	***
$CaCl_2$	***	***
$BaCl_2$	***	***
$MnCl_2$	***	***
$CoCl_2$	***	***
$NiCl_2$	***	***
$CuCl_2$	***	***
$ZnCl_2$	***	***
$NiSO_4$	***	***
$ZnSO_4$	***	***

Table S1B. Holm-Bonferroni statistical analysis of **Figure 3A** between PFE + 5 mM metal in absence and presence of EDTA. Statistical significance is explained in **Table S1A**.

metal	PFE + 5 mM metal + EDTA vs. PFE + 5 mM metal – EDTA		
	p	$\Delta_{activity}$	interpretation
$MgCl_2$	0.009 (**)	-4%	no recovery
$CaCl_2$	<0.001 (***)	+13%	moderate recovery
$BaCl_2$	<0.001 (***)	+9%	modest recovery
$MnCl_2$	<0.001 (***)	+24%	significant recovery
$CoCl_2$	0.316 ns	-1%	no recovery
$NiCl_2$	<0.001 (***)	+5%	modest recovery
$CuCl_2$	<0.001 (***)	+51%	significant recovery

$ZnCl_2$	<0.001 (***)	+25%	significant recovery
$NiSO_4$	0.003 (**)	-5%	no recovery
$ZnSO_4$	<0.001 (***)	+54%	significant recovery

Δ_{act}= activity$_{(+EDTA)}$ – activity$_{(–EDTA)}$

Table S2. Mono-exponential decay constant (t_1) and apparent IC_{50} values for metal-induced PFE deactivation. All metals were in the form of chlorides, unless otherwise stated; *n.d.* means not determined.

	t_1 / mM	apparent IC_{50}
Mg^{2+}	21.8 ± 6.4	15.1 ± 4.4
Ca^{2+}	21.0 ± 9.7	14.6 ± 6.7
Ba^{2+}	19.5 ± 6.7	13.5 ± 4.7
Mn^{2+}	17.8 ± 8.3	12.4 ± 5.8
Co^{2+}	18.6 ± 13.9	12.9 ± 9.6
Ni^{2+}	44.5 ± 10.8	30.8 ±7.5
Cu^{2+}	0.6	0.4
Zn^{2+}	15.1	10.5
$NiSO_4$	*n.d.*	*n.d.*
$ZnSO_4$	3.2 ± 0.2	2.3 ± 0.1

Table S3. Relative catalytic activity of several lipases and esterases in presence of divalent metal ions and comparison with our data (last column). In red color are values that match our data; columns highlighted in beige denote overall agreement with our data; *n.d.* means not detected; - means not measured; values with ~ were estimated from graphs.

	LipB[1], a	AMS8 lipase[2]	KB-Lip[3]	LipP[4], b	rPML[5]	SIK W1 lipase[6], c	Esterase *B. subtilis*[7]	EstN3[8]	Est10[9]	EstDR4[10]	Acetylxylan Esterase[11]	BaCE[12]	In this work
Co^{2+}	84 ± 2%	~60%	5%	58%	*n.d.*	98 ± 3%	-	-	-	~35%	30 ± 2%	~35%	80%
Ni^{2+}	88 ± 2%	~35%	15%	-	*n.d.*	-	-	68 ± 3%	-		10 ± 7%	~42%	90%
Mg^{2+}	183 ± 3%	~95%	40%	90%	*n.d.*	124 ± 1%	35%	94 ± 12 %	96 ± 8%	~105%	123 ± 8%	~85%	83%
Ca^{2+}	202 ± 2%	~80%	560%	58%	100%	665± 30%	86%	89 ± 3%	130 ± 5	~70%	24 ± 9%	~90%	86%
Mn^{2+}	53 ± 1%	~30%	120%	29%	*n.d.*	130 ± 3%	30%	74 ± 2%	100 ± 8%	~38%	101.2 ± 8%	~70%	68%
Ba^{2+}	180 ± 2%	-	-	-	-	-	-	-	131 ± 1%	-	-	~70%	77%
Cu^{2+}	32 ± 2%	*n.d.*	4%	4%	*n.d.*	102 ± 9%	19%	*n.d.*	94 ± 9%	~30%	13 ± 8%	~23%	22%
Zn^{2+}	52 ± 1%	*n.d.*	2%	9%	*n.d.*		16%	40 ± 10%	100 ± 2%	~22%	8 ± 4%	~30%	61%
EDTA	82 ± 3%	-	*n.d.*	109%	-	-	60%*	-	117 ± 7%	-	-	-	100 %

[a]metal and EDTA concentrations were 10 mM and 1 mM, respectively.
[b]metal and EDTA concentrations were 10 mM and 5 mM, respectively.
[c]metal concentration was 2 mM.
*EDTA concentration was 1 mM.

Table S4. Residues of PFE exhibiting the highest predicted affinity for divalent metal ion binding based on *in silico* analysis.

metal	Residue names		
Mg^{2+}	Asp68	Asp70	Thr71
Ca^{2+}	Asp33	Asp68	Asp70
Ba^{2+}	Glu40	Ser43	Asp248
Mn^{2+}	Asp70	Thr71	Asp74
Co^{2+}	Asp70	Asp74	Asp130
Ni^{2+}	Asp68	Asp70	Thr71
Cu^{2+}	Ser94	His251	His218

Table S5. Representative residual activity of PFE (10 nM) incubated at 37°C in presence of 5 mM divalent metal ions up to 48 h and residual activity was measured at 27°C using *p*NPB substrate (0.75 mM); error bars represent standard deviation from triplicates. Samples that show early activation and pronounced early deactivation are marked in green and orange, respectively. n.d. means not detected.

	**% activity 2 h**	**% activity 4 h**	**% activity 24 h**
Native PFE	140 ± 3.0	108 ± 2.0	96 ± 2.0
Mg^{2+}	146 ± 2.0	148 ± 2.0	97 ± 1.0
Ca^{2+}	117 ± 3.0	135 ± 2.0	82 ± 3.0
Ba^{2+}	181 ± 1.0	177 ± 1.4	116 ± 1.7
Mn^{2+}	111 ± 1.0	72 ± 0.6	*n.d.*
Co^{2+}	7.8 ± 0.6	3.0 ± 2.3	*n.d.*
Ni^{2+}	82 ± 1.0	72 ± 1.0	*n.d.*
Cu^{2+}	12.9 ± 8.3	7.9 ± 5.3	*n.d.*
Zn^{2+}	9.1 ± 2.1	*n.d.*	*n.d.*

6. References

[1] Z. Hu, L. Jiao, X. Xie, L. Xu, J. Yan, M. Yang, Y. Yan,"Characterization of a New Thermostable and Organic Solution-Tolerant Lipase from Pseudomonas fluorescens and Its Application in the Enrichment of Polyunsaturated Fatty Acids", *International Journal of Molecular Sciences* **24**, (2023): 8924.

[2] M. Ganasen, N. Yaacob, R. N. Z. R. A. Rahman, A. T. C. Leow, M. Basri, A. B. Salleh, M. S. M. Ali,"Cold-adapted organic solvent tolerant alkalophilic family I.3 lipase from an Antarctic Pseudomonas", *International Journal of Biological Macromolecules* **92**, (2016): 1266-1276.

[3] N. Rashid, Y. Shimada, S. Ezaki, H. Atomi, T. Imanaka,"Low-temperature lipase from psychrotrophic *Pseudomonas* sp. strain KB700A", *Applied and Environmental Microbiology* **67**, (2001): 4064-4069.

[4] D.-W. Choo, T. Kurihara, T. Suzuki, K. Soda, N. Esaki,"A Cold-Adapted Lipase of an Alaskan Psychrotroph, Pseudomonas sp. Strain B11-1: Gene Cloning and Enzyme Purification and Characterization", *Applied and Environmental Microbiology* **64**, (1998): 486-491.

[5] K. Amada, M. Haruki, T. Imanaka, M. Morikawa, S. Kanaya,"Overproduction in Escherichia coli, purification and characterization of a family I.3 lipase from Pseudomonas sp. MIS38", *Biochimica et Biophysica Acta (BBA) - Protein Structure and Molecular Enzymology* **1478**, (2000): 201-210.

[6] L. Young Phil, C. Guk Hoon, R. Joon Shick,"Purification and characterization of Pseudomonas fluorescens SIK W1 lipase expressed in Escherichia coli", *Biochimica et Biophysica Acta (BBA) - Lipids and Lipid Metabolism* **1169**, (1993): 156-164.

[7] A. Asoodeh, T. Ghanbari,"Characterization of an extracellular thermophilic alkaline esterase produced by *Bacillus subtilis* DR8806", *Journal of Molecular Catalysis B: Enzymatic* **85-86**, (2013): 49-55.

[8] M. K. Yadav, R. Ranjan, P. Verma, R. Sharma,"Discovery and characterization of an enantioselective family VIII esterase from effluent treatment plant sludge metagenome", *Scientific Reports* **16**, (2025): 252.

[9] G. Wu, G. Wu, T. Zhan, Z. Shao, Z. Liu,"Characterization of a cold-adapted and salt-tolerant esterase from a psychrotrophic bacterium Psychrobacterpacificensis", *Extremophiles* **17**, (2013): 809-819.

[10] Y. Zhang, W. Lu, J. Wang, M. Chen, W. Zhang, M. Lin, Z. Zhou, Z. Liu,"Characterization of EstDR4, a Novel Cold-Adapted Insecticides-Metabolizing Esterase from Deinococcus radiodurans", *Applied Sciences* **11**, (2021): 1864.

[11] J. Lu, H. Bi, R. Zhang, X. Liu, B. Wang, J. Wu, J.-K. Lee, V. C. Kalia, C. Gong,"Pesticide Biodegradation Catalyzed by a Cold-Adapted Acetylxylan Esterase Identified from a Metagenome-Assembled Genome", *Journal of Agricultural and Food Chemistry* **74**, (2026): 8187-8197.

[12] Y.-J. Zhang, C.-S. Chen, H.-T. Liu, J.-L. Chen, Y. Xia, S.-J. Wu,"Purification, identification and characterization of an esterase with high enantioselectivity to (S)-ethyl indoline-2-carboxylate", *Biotechnology Letters* **41**, (2019): 1223-1232.
